# Reevaluation of data interpretation in study on pre-excitation risk assessment

**DOI:** 10.1093/europace/euae119

**Published:** 2024-05-04

**Authors:** José Nunes de Alencar

**Affiliations:** Cardiology, Instituto Dante Pazzanese de Cardiologia, Avenida Dr. Dante Pazzanese No. 500, Vila Mariana, São Paulo 04012-091, Brazil

I have reviewed the recent publication by Jemtrén et al. titled ‘Risk assessment in patients with symptomatic and asymptomatic pre-excitation’^1^ with great interest. This study offers important insights into the use of non-invasive exercise stress tests (EST) as an index test in contrast to the invasive measurement of accessory pathway effective refractory period (APERP) and the shortest pre-excited RR interval (SPERRI) in predicting the risk of arrhythmic events. In their analysis, the authors reported the sensitivity, specificity, positive predictive value (PPV), negative predictive value (NPV), and accuracy of a low-risk EST for excluding patients with SPERRI/APERP ≤ 250 ms as 40%, 91%, 87%, 51%, and 60%, respectively.

What drew my attention was the authors’ conclusion stating that ‘Sudden loss of pre-excitation during EST has a low NPV in excluding high-risk accessory pathways (APs).’ This prompted me to examine the data more closely, and I discovered a significant inconsistency in data interpretation that I will clarify in the following paragraphs.

As the sensitivity and specificity calculated by Jemtrén et al. were 40% and 91%, respectively, this suggests that the contingency table constructed for the study was the one shown in *Table 1*. This is confirmed in ‘Table 3’ of Jemtrén's paper. With this set-up, the PPV would be 87%, and the NPV was 51%.

**Table 1:** Jemtrén’s contingency table. In this configuration, sensitivity is 40.2% and specificity 91%.

	Low risk (APERP/SPERRI > 250 ms)—Disease present	High risk (APERP/SPERRI < 250 ms)—Disease absent
Test positive (sudden loss of pre-excitation)	39—TP	6—FP
Test negative (no sudden loss of pre-excitation)	58—FN	61—TN

PPV is the probability that the disease is present when the test is positive, and NPV is the probability that the disease is not present when the test is negative.^2^


$$Positive\;predictive\;value=\;\frac{True\;positives\;(those\;with\;sudden\;loss\;of\;pre\text{-}excitation\;that\;had\;low\text{-}\;risk\;APs)}{All\;positives\;(all\;patients\;with\;sudden\;loss\;of\;pre\text{-}excitation)}=\;\frac{39}{45}\;=\;86.6\text{\%}.$$

Negativepredictivevalue=Truenegatives(thosewithnosuddenlossofpre-excitationthathadhigh-riskAPs)Allnegatives(allpatientswithnosuddenlossofpre-excitation)=61119=51.2%.

Since Jemtrén defined ‘true positives’ as those with a sudden loss of pre-excitation who had a low-risk AP, they consequently and inadvertently defined ‘diseased’ individuals as those who had a low-risk AP, a mistake also made by other authors in the same field.^3,4^ Consequently, the PPV of 87% actually implies a high likelihood that a sudden loss of pre-excitation indicates a low-risk AP, as 39 of the 45 positive results confirmed low-risk status.

The generated NPV of 51% indicates that the absence of the finding cannot confirm ‘absence of disease’, that was defined by Jemtrén as those who have high-risk APs; therefore, the absence of sudden loss of pre-excitation cannot confirm high risk APs. That said, the statement in conclusion that ‘Sudden loss of pre-excitation during EST has a low NPV in excluding high-risk APs’ is conceptually incorrect.

Furthermore, from the contingency table created by the authors, the positive likelihood ratio (LR+) is 4.49 (95% CI: 2.02–10.00), indicating that a sudden loss of pre-excitation (positive result) multiplies the risk by four to five that a positive test signifies a low-risk pathway (diseased)—a reasonable (not astonishing, but reasonable) result.^5^

Had the authors constructed the contingency table with true positives defined as the absence of pre-excitation loss and diseased patients as those with high-risk accessory pathways, the resulting table would be different (*Table 2*). In this scenario, the swapped results would show a sensitivity of 90.2%, specificity of 40%, PPV of 51%, and NPV of 87%. In this case, the negative likelihood Ratio (LR-) of 0.22 (95% CI: 0.10–0.50) would indicate that a negative test result—meaning the presence of pre-excitation loss—makes the presence of a high-risk AP about 4–5 times less likely compared with if this test result had not been observed.

**Table 2:** Alternate contingency table, generated under the assumption that true positives are defined as cases with no loss of pre-excitation and diseased individuals as those with high-risk accessory pathways. In this configuration, the sensitivity, and specificity would be 91% and 40.2%, respectively, with a swap in the PPV and NPV values; the NPV would now be 87%. Despite these changes, the interpretation remains consistent: in this study, the loss of pre-excitation (negative test) still indicates a high predictive value for identifying low-risk accessory pathways (absence of disease).

	High risk (APERP/SPERRI < 250 ms)—Disease present	Low risk (APERP/SPERRI > 250 ms)—Disease absent
Test positive (no sudden loss of pre-excitation)	61—TP	58—FP
Test negative (sudden loss of pre-excitation)	6—FN	39—TN

I conclude that the authors of the study misinterpreted what constitutes a positive or negative test and the definition of those with and without the condition, leading to confusion, and incorrect conclusions. While their intentions were undoubtedly aligned with advancing our understanding, it appears that a more thorough analysis of the data yields the opposite of what was initially posited. According to these data, the sudden loss of ventricular pre-excitation decreases the likelihood that the patient has a high-risk pathway.

## Data Availability

There are no new data associated with this article.
